# Necrotizing Cellulitis of the Finger Caused by Ring Embedment Leading to Amputation: A Case Report and Literature Review

**DOI:** 10.7759/cureus.98746

**Published:** 2025-12-08

**Authors:** Shreya Guha, Manya Bali, Zohaer Muttalib, Eldo Frezza

**Affiliations:** 1 Medical School, California Northstate University College of Medicine, Elk Grove, USA; 2 Surgery, California Northstate University College of Medicine, Elk Grove, USA

**Keywords:** digit amputation, infection, ischemia, necrotizing cellulitis, oxygen therapy, pressure ulcer, reperfusion injury, tissue necrosis

## Abstract

Constrictive ring embedment can cause progressive ischemia, tissue necrosis, and infection. Although embedded rings have been reported, digital amputation as a direct result is rarely documented.

The authors present the case of a 62-year-old woman who presented to the emergency department with a five-day history of right ring finger cellulitis secondary to prolonged ring erosion extending to the bone. She reported pain, fever, and weakness, and was tachycardic with signs of sepsis. A physical exam revealed crepitus, though radiographs showed no subcutaneous air. Given the severity of ischemic tissue damage and the risk of reperfusion injury, the patient was transferred to the orthopedic service for digital amputation.

The Braden scale, a measurement tool for pressure ulcer risk, identified her as high-risk for pressure ulcers, emphasizing the need for early intervention. Prolonged ring pressure had led to capillary occlusion, ischemia, and necrosis, creating an environment for bacterial infection. Ischemia-driven hypoxia further impaired bacterial clearance and wound healing, while reperfusion injury complicated management.

This case highlights the importance of early recognition and intervention in ring-related ischemic injuries. Clinicians should be aware of the potential for severe vascular compromise, infection, and reperfusion injury in constrictive ring embedment.

## Introduction

Necrotizing cellulitis (NC) is a rare but serious bacterial infection of the skin that can lead to tissue destruction, severe inflammation, and potential systemic spread. NC most commonly presents in patients with pre-existing conditions that compromise immunity or tissue perfusion. Common risk factors for NC include diabetes mellitus, peripheral vascular disease, chronic kidney disease, immunosuppression, chronic wounds or ulcers, recent surgeries, and IV drug use, among other factors. Necrotizing fasciitis is rapidly progressive and is characterized by high morbidity and mortality rates. Common presenting symptoms include localized pain (93.8%), swelling (80.6%), and erythema (79.5%), often accompanied by systemic signs such as fever (55%) and hypotension in advanced stages. The condition carries a mortality rate ranging from 20% to 40%, with delays in diagnosis significantly increasing the risk of death [1]. NC is most commonly caused by *Clostridium* spp., Group A *Streptococcus*, and mixed anaerobic and facultative bacterial species [2]. It can occur through a variety of mechanisms, including a break in the skin, puncture wounds, and ischemia, which can all lead to necrosis and gangrene of the affected area. Necrotizing fasciitis can develop rapidly, often within 12-24 hours, and presents with spreading erythema, warmth, and swelling in the affected area. It progresses with blistering, bullae, and skin necrosis. It is also associated with severe pain that seems out of proportion to visible signs and quickly worsens over the following few days [3,4]. Without prompt antibiotics or surgical debridement, sepsis and death can occur in the following one to two days after symptoms.

Additionally, crepitus can sometimes be heard, indicating a gas-forming bacterium such as *Clostridium* spp. Dark discoloration and foul-smelling discharge are also possible. Systemic symptoms such as high fever, tachycardia, chills, and hypotension may accompany local symptoms. If testing and therapy are not begun adequately, septic shock may occur, as well as rapid necrosis and toxic shock syndrome. Diagnostic tests to identify NC include imaging studies, blood tests, and tissue cultures or Gram stains. Although there is no single definitive diagnostic guideline for necrotizing fasciitis, there are recognized clinical tools and criteria to aid diagnosis. A widely used tool to aid in the diagnosis of necrotizing fasciitis is the Laboratory Risk Indicator for Necrotizing Fasciitis (LRINEC), which incorporates C-reactive protein levels, white blood cell count, hemoglobin, sodium, creatinine, and glucose levels [5]. When used in conjunction with imaging studies and clinical evaluation, this scoring system can help identify necrotizing fasciitis in high-risk patients. Treatment includes surgical debridement, broad-spectrum antibiotics, and IV fluids [6,7]. 

NC most commonly develops following a disruption of the integumentary barrier, permitting the translocation of pathogenic microorganisms, typically polymicrobial flora including anaerobes and facultative anaerobes, into subcutaneous tissues. In high-risk ischemic regions, such as digits or extremities with compromised vascular supply, the susceptibility to infection increases significantly due to hypoperfusion. Ischemia results in diminished oxygen tension, impaired neutrophil function, and reduced delivery of essential metabolic substrates such as glucose, creating an ideal microenvironment for anaerobic bacterial proliferation. This facilitates the production of bacterial exotoxins and proteolytic enzymes. This promotes rapid tissue necrosis and fascial dissection. Additionally, ischemic tissues exhibit reduced immune surveillance and delayed wound healing, exacerbating the risk of infection. The resulting inflammatory cascade and tissue degradation can precipitate systemic inflammatory response syndrome (SIRS) and sepsis. Prompt recognition and intervention, including surgical debridement, hemodynamic support, and broad-spectrum antimicrobial therapy, are imperative to mitigate morbidity and mortality in patients [8].

Although NC is a rare medical concern in the United States, accounting for around 1,000 cases yearly, proper management and treatment are necessary to prevent significant necrosis, tissue damage, and possible amputation [9]. In accordance with the practice guidelines for the diagnosis and management of skin and soft tissue infections by the Infectious Diseases Society of America, common medical management treatments include broad-spectrum antibiotics, targeted antibiotics, IV fluid resuscitation therapies, and vasopressor support to help patients with this infection [9]. Close and regular monitoring is needed to prevent serious symptoms in patients with NC. This case is a unique presentation of a patient who presents with severe pain in her right digit after wearing a very tight ring. This may have led to ischemia and the growth of bacteria, leading to NC.

## Case presentation

**Figure 1:**
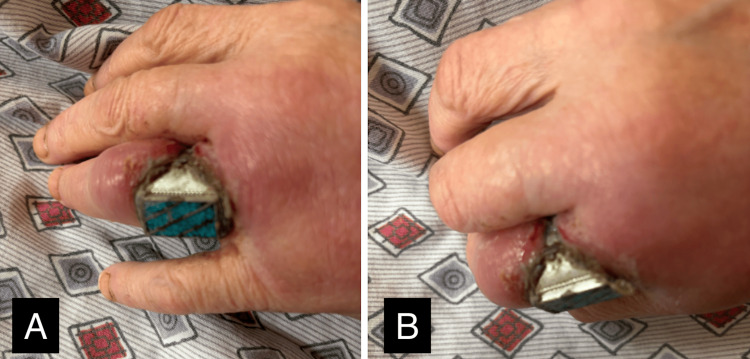
Silver ring embedded on the patient’s right hand A, B: evidence of erosion through skin and tissue.

A 62-year-old female presented to the emergency department with tissue and skin necrosis of the right ring finger. She was wearing a silver ring that had eroded through the skin of her finger (Figure 1). There had been pain and swelling of the finger for five days, and she came to the emergency department when she could no longer feel or move the finger. At arrival, the patient presented with fever, weakness, and tachycardia. The initial blood pressure was 105/60 mmHg, her white blood cell count was elevated at 17,000/mm³, and serum lactate was 5.2 mmol/L. These findings were suggestive of tissue hypoxia secondary to sepsis from infection at the site of the finger wound. Blood cultures were taken, and she was promptly administered two 1000 cc bags of IV 0.9% saline as well as Zosyn (piperacillin and tazobactam) 4.5 g infused over six hours. Following this, her blood pressure increased to 120/75 and remained stable during her ED stay. Her current medication list included metformin, lisinopril, atorvastatin, and alprazolam (Xanax). Laboratory values and vital signs are summarized in Table 1.

**Table 1:** Laboratory values and vital signs

Test	First Reading	Second Reading	Reference Range
White Blood Cell Count	17,000/mm^3^	-	4500-11,000/mm^3^
Serum Lactate	5.2 mmol/L	-	0.5-2.2 mmol/L
Blood Pressure	105/60 mmHg	120/75 mmHg	120/80 mmHg

**Figure 2:**
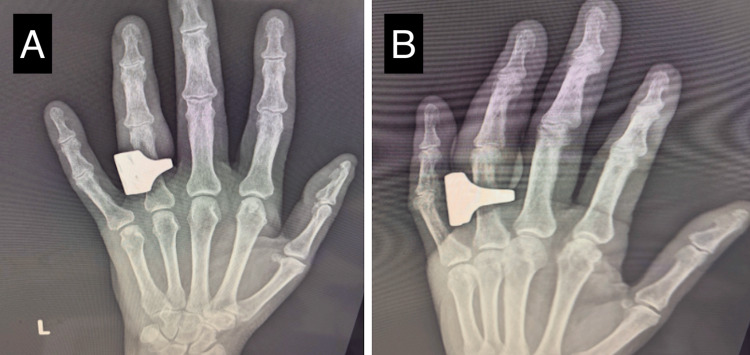
X-ray images of the patient’s right hand with the embedded ring Radiograph of the right hand depicting ring embedment, unremarkable for fractures or crepitus. A: posteroanterior; B: oblique

On closer examination, the wound appeared ischemic and malodorous. When the ring was gently moved, bone was visible underneath. Crepitus was present on physical exam, but X-rays did not show any pockets of air (Figure 2). The ring was unable to be removed in the ED, as there was no space to insert a cutting instrument. The patient was transferred to an orthopedic hand surgeon, where the ring was cut in the operating room (OR). Duplex ultrasound revealed no distal perfusion of the finger following removal of the ring, so the right ring finger was amputated at the third (distal) phalanx. Blood cultures were later positive for *Streptococcus A*.

After three days, she was discharged home with recommendations for home nursing to manage the dressing of the wound.

## Discussion

Ring erosion to the bone of a finger involves continuous pressure, edema, and ischemia. Prolongation of the repetitive trauma can lead to infection, cellulitis in the case of our patient, and require amputation. Previous reports and reviews have not discussed amputation of a digit as a consequence of ring embedment [10]. Our patient presents a unique case of ring embedment that required digital amputation due to an inability to remove the ring and risk of reperfusion injury. 

Pressure ulcers

The swelling of the finger may be secondary to pressure ulcers, which involve continuous pressure over bony prominences or constricted areas, which can lead to the disruption of local blood flow [11]. As seen in this case, this can lead to ischemia and necrosis of the localized soft skin and tissue [11]. Such ischemia can also create an anaerobic environment, providing ideal conditions for bacterial colonization and infection, as seen with the patient’s cellulitis. 

Evidence suggests that even mild pressure over long durations of time can induce tissue hypoxia due to capillary bed occlusion at pressures above 32 mmHg [11]. In this case, the ring, acting as the constrictive force, allowed for the exacerbation of local ischemia and bacterial entry into the epidermis. A case report outlining the partial embedding of a ring around a finger outlines how digital swelling can be exacerbated by the presence of the ring, leading to venous congestion and, therefore, arterial compromise [12]. 

In evaluating pressure ulcers, there are three risk assessment scales: Waterlow, Norton, and Braden [13-15]. The higher the score, the greater the susceptibility for an individual to experience a pressure ulcer. The Braden scale is most commonly used due to its ease and incorporation of risk factors. The Braden scale assesses pressure ulcer risk by evaluating sensory perception, moisture, activity, mobility, nutrition, and friction/shear [11,15]. 

Our patient, presenting with diabetes, ischemic tissue, and immobility in the affected finger, was at increased risk for localized pressure ulcers and poor wound healing. The patient could not feel or move the affected finger, indicating complete sensory loss in that area. The wound was malodorous and ischemic, suggesting it was frequently moist due to infection and necrosis. The patient had diabetes, hyperlipidemia, and hypertension, which suggests potential dietary concerns affecting healing and overall nutritional status. Along with other factors of sepsis and weakness, we predict the patient to have a score of 12, placing the patient in the high-risk category for pressure ulcers. The lack of perfusion compounded the risk of tissue breakdown, emphasizing the importance of early intervention and pressure offloading strategies. In future practice, incorporating Braden scale assessments in similar cases can help predict and mitigate pressure-related complications.

Ischemia

Oxygen is an essential factor in tissue repair and infection control. Hypoxia is often seen in infected tissues and can impair neutrophil function, therefore reducing effective bacterial clearance and delaying wound healing [16]. A combination of compromised oxygenation due to a loss of local blood flow from mechanical pressure and infection-based inflammation likely reflects the lack of perfusion in this patient’s presentation.

Evidence suggests that hyperbaric oxygen therapy (HBOT) is an innovative approach to enhance wound healing in the case of severe infection management. HBOT is a technique that increases oxygen solubility in plasma, thereby promoting collagen synthesis, angiogenesis, and bacterial killing by neutrophils [16]. In terms of microbial infections, evidence suggests that HBOT can be considered for non-healing wounds in an attempt to break bacterial biofilms [16]. Yet, the most important application of antimicrobial activity for HBOT can be applied to necrotizing soft tissue infections. Anaerobic bacteria are killed at a much higher rate when HBOT is used, which can decrease levels of inflammatory factors, TNF-α, IL-6, and IL-10 [16]. This can lead to improved oxygen flow and prevent the need for amputation.

HBOT has also been shown to play a role in angiogenesis and revascularization with the production of vascular endothelial growth factor (VEGF), platelet-derived growth factor (PDGF), and fibroblast growth factor (FGF) [17]. 

Studies show that HBOT, which is used as adjunct therapy for diabetic foot ulcers, can provide faster healing and better outcomes than patients not treated with HBOT [18]. As wound hypoxia remains a strong risk factor for patients with non-healing wounds, increasing oxygen on a cellular level is a tactic to help upregulate growth factors, reduce inflammation, increase collagen and blood vessel synthesis, and eliminate bacteria. Studies continue to depict lower major amputation rates for diabetic foot ulcers with the use of adjunctive HBOT [19]. 

Ischemia played a central role in this case, as the prolonged constriction from the ring led to necrosis. The inability to perfuse the digit after ring removal confirmed irreversible tissue damage. This highlights the need for early recognition of ischemic changes in similar cases, where prolonged vascular compromise can lead to necrotic progression. Clinically, practitioners should prioritize prompt assessment of circulation and tissue viability in patients with constrictive injuries to improve outcomes and minimize amputation risk.

Vascular reperfusion injuries

Post amputation, the revascularization of ischemic tissue can trigger oxidative stress and inflammatory processes, which can exacerbate cellular damage. Such reperfusion injury involves the formation of reactive oxygen species (ROS), microvascular vasoconstriction, and activation of neutrophils to release proinflammatory cytokines, which further damage tissue [20]. 

Management of reperfusion injuries involves adequate hydration pre- and postoperatively to help maintain flow and reduce renal damage from toxic metabolites. Additionally, the use of bicarbonate or other agents can reduce acidosis that occurs during reperfusion [20]. 

The prevention of vascular reperfusion injury includes ischemic pre- or post-conditioning, which involves organ exposure to small durations of ischemia and limited reperfusion of the organ in order to prevent ROS formation [20].

Reperfusion injury was a major concern in this case, influencing the decision not to cut the ring in the emergency setting. Sudden restoration of blood flow to ischemic tissues can lead to significant oxidative stress and inflammation, exacerbating tissue damage. This case illustrates the delicate balance between timely intervention and the potential harms of reperfusion injury. Future clinical guidelines should consider risk stratification for reperfusion injury, especially in patients with chronic ischemic insults, to optimize both surgical timing and patient outcomes.

## Conclusions

This rare case of ring-induced NC necessitating amputation underscores the need for prompt recognition of ischemic injuries. Risk assessment tools like the Braden scale may aid prevention, though HBOT's role requires further study. Clinicians should maintain a high suspicion for vascular compromise in constrictive ring injuries to avert irreversible damage.
